# Adipokine-mediated metabolic responses across BMI in acute myocardial infarction

**DOI:** 10.1186/s12872-026-06140-w

**Published:** 2026-06-20

**Authors:** Sunita Sunita, Mas Rizky A. A. Syamsunarno, Mohammad Rizki Akbar, Annisa Dewi Nugrahani, Nova Sylviana, Gaga Irawan Nugraha, Mohammad Ghozali, Tri Hanggono Achmad

**Affiliations:** 1 Doctoral Study Program Faculty of Medicine, Universitas Padjadjaran, Bandung, West Java Indonesia; 2 Department of Biomedical Sciences, Faculty of Medicine, Universitas Padjadjaran, Bandung, West Java Indonesia; 3 Cardiometabolic Working Study Group, Faculty of Medicine, Universitas Padjadjaran, Bandung, West Java Indonesia; 4 Department of Cardiology and Vascular Medicine, Faculty of Medicine, Universitas Padjadjaran- Hasan Sadikin General Hospital, West Java, Indonesia; 5 Department of Medical Laboratory Technology, Poltekkes Kemenkes Bengkulu, Bengkulu, Indonesia

**Keywords:** Acute myocardial infarction, Adipokines, Adiponectin, Growth differentiation factor 15, Obesity

## Abstract

**Background:**

Adipose tissue secretes adipokines, bioactive proteins that regulate inflammation and cardiometabolic processes. During acute myocardial infarction (AMI), adipokine profiles may vary according to body mass index (BMI), yet this association remains underexplored.

**Aims:**

To compare circulating adiponectin, fibroblast growth factor 21 (FGF21), growth differentiation factor 15 (GDF15), and interleukin-6 (IL-6) between non-obese and obese patients with AMI.

**Methods:**

In this cross-sectional study, 105 patients with AMI were stratified as non-obese (BMI < 25 kg/m², *n* = 62) or obese (BMI ≥ 25 kg/m², *n* = 43). Serum adiponectin, GDF15, and IL-6 were measured by Luminex, and FGF21 was measured by ELISA. Clinical variables and lipid indices were compared between BMI groups, and correlation analyses were performed.

**Results:**

Although myocardial injury levels were comparable between the two groups, non-obese patients had significantly higher serum levels of adiponectin, FGF21, GDF15, and IL-6 than obese patients (all *p* < 0.05). In all patients, adiponectin was negatively correlated with total cholesterol, LDL-c, and triglycerides, and positively correlated with HDL-c; however, these associations were not consistently observed within BMI subgroups after stratification and FDR correction. GDF15 and IL-6 also showed inverse correlations with selected lipid parameters in all patients.

**Conclusion:**

Non-obese AMI patients exhibited higher levels of adiponectin, FGF21, GDF15, and IL-6 compared to obese AMI patients. These adipokine profiles were associated with lipid parameters, notably showing inverse correlations between adiponectin and GDF15 with total cholesterol and LDL-c. These findings highlight the association between adiposity and early metabolic responses in AMI across BMI categories.

**Supplementary Information:**

The online version contains supplementary material available at https://doi.org/10.1186/s12872-026-06140-w.

## Introduction

Promoting health and well-being requires addressing modifiable cardiovascular risk factors, especially obesity, through preventive interventions and equitable access to cardiac care. These strategies are essential to reducing the occurrence, complications, and early mortality related to AMI, one of the most serious manifestations of cardiovascular diseases **(**CVD) [1]. Cardiovascular disorders continue to be the foremost cause of death worldwide, with AMI contributing substantially to morbidity and mortality. AMI typically results from the sudden rupture of atherosclerotic plaques, leading to ischemia, myocardial necrosis, and a cascade of inflammatory and metabolic disturbances [2–4].

Obesity is a major determinant of AMI risk and commonly coexists with diabetes mellitus, hypertension, and dyslipidemia [5–7]. Yet BMI does not capture the full biological complexity of adiposity. The frequently discussed “obesity paradox,” whereby overweight or obese patients sometimes experience better short-term outcomes after AMI, suggests that body mass alone may not explain early post-infarction physiology [8–10].

This paradox suggests that factors beyond total adipose tissue volume, such as fat distribution, adipose tissue quality, and adipokine secretion, may influence cardiovascular outcomes [11–13]. Moreover, adipose tissue appears to have a more nuanced role in AMI pathophysiology. In addition to its contribution as a major risk factor, the early activation of adipose-related inflammatory and metabolic pathways may contribute to heterogeneity in circulating biomarker profiles during the initial phase of AMI. This dualistic nature of adipose tissue emphasizes the complexity of its involvement in both the development and clinical trajectory of AMI.

Adipose tissue characteristics, encompassing its quality, anatomical distribution, and endocrine activity, provide a more meaningful understanding of cardiometabolic risk than BMI alone[14]. Adipokines are generally defined as cell-signaling proteins secreted by adipose tissue and are closely related to low-grade inflammation and various cardiometabolic pathologies [15]. They play central roles in modulating inflammation, lipid metabolism, and endothelial function [16–18]. A growing body of evidence emphasizes their importance in metabolic homeostasis and cardiovascular health [14, 18–23].

Our earlier work highlights that obesity was consistently linked with altered circulating adipokines, reinforcing the concept that adipose tissue dysfunction extends cardiometabolic risk beyond conventional anthropometry [14]. Among the candidate mediators, adiponectin is notable for anti-atherogenic and insulin-sensitizing actions and has been linked to prognosis in AMI and heart failure [24–27]. FGF21 coordinates glucose and lipid metabolism and may rise during myocardial ischemia as part of an adaptive stress program [28, 29]. GDF15 is a cytokine induced under cellular stress conditions that has been linked to inflammation, redox imbalance, and mitochondrial dysfunction, and is increasingly recognized as a cardiovascular biomarker [30]. IL-6 occupies a more complex position, functioning as both an inflammatory amplifier and a context-dependent mediator of repair and metabolic regulation [31, 32].

Taken together, we postulated that BMI influences the early adipokine landscape during AMI. We therefore compared adiponectin, FGF21, GDF15, and IL-6 between non-obese and obese patients with AMI and explored their relationships with lipid parameters to better define the metabolic response associated with acute myocardial injury.

## Methods and materials

### Study design and participants

This single-center cross-sectional observational study was performed at Hasan Sadikin General Hospital, Bandung, Indonesia, between February and November 2023. We screened 150 consecutive patients presenting with AMI. Eligible participants had ST-elevation myocardial infarction (STEMI) or non-ST-elevation myocardial infarction (NSTEMI) diagnosed by a cardiologist using standard clinical presentation, electrocardiographic findings, and biochemical evidence of myocardial injury. The biomarker panel was selected to capture adipokines repeatedly implicated in obesity-related cardiometabolic dysfunction in our previous study [14].

STEMI was defined by ischemic symptoms, Elevated levels of cardiac troponin I are determined based on the established test threshold, with a cut-off value of ≥ 0.02 ng/mL, and new ST-segment elevation at the J-point in at least two contiguous leads (≥ 0.1 mV in most leads; in leads V2–V3, ≥ 0.2 mV in men and ≥ 0.15 mV in women). NSTEMI was defined as AMI without persistent ST-segment elevation, accompanied by ST depression, T-wave inversion, or transient ST elevation together with elevated troponin concentrations [33].

Exclusion criteria included patients with unstable angina pectoris, concurrent stroke, incomplete clinical or laboratory data, and those who died. After excluding 45 patients due to death (*n* = 15), unstable angina (*n* = 10), or incomplete data (*n* = 20), a total of 105 AMI patients were included in the final analysis. Participants were stratified using the Asia-Pacific BMI threshold into non-obese (BMI < 25 kg/m², *n* = 62) and obese (BMI ≥ 25 kg/m², *n* = 43) groups [34].

**Fig. 1:**
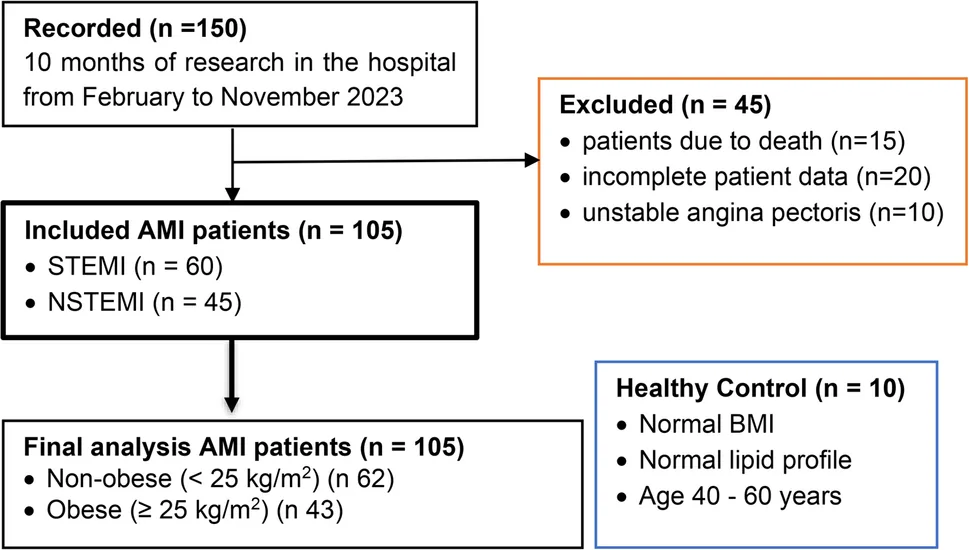
Flow chart showing patient inclusion and exclusion criteria and final sample size determination, including evaluation of 10 healthy controls

Ten healthy individuals were recruited to establish reference ranges for circulating adipokine levels. These controls were not included in hypothesis testing or group comparisons. Healthy participants were adults without known metabolic, cardiovascular, renal, or hepatic disease, confirmed by clinical evaluation and basic laboratory screening. All control subjects underwent at least 8 h of fasting prior to sample collection. As shown in Fig. 1, control values were used solely to define reference levels for adipokine visualization.

## Data collection

### Anthropometric and clinical measurements

Body weight and height were obtained using calibrated instruments (OMRON HBF-214, Singapore; ADE MZ10042, Germany). Anthropometric measurements were collected after clinical stabilization, once patients had regained adequate mobility and were able to undergo body weight, height, waist circumference, and body composition assessment safely. Waist circumference was measured midway between the lowest rib and iliac crest using a non-stretch tape, and the waist-to-height ratio (WHtR) was calculated as waist circumference divided by height and classified as normal (0.40–0.49) or high (≥ 0.50). Body fat percentage was assessed by bioelectrical impedance analysis (OMRON BMC-500; OMRON Healthcare Co., Ltd., Kyoto, Japan), and blood pressure was measured with a digital sphygmomanometer from the same manufacturer.

Acute myocardial infarction risk factors were assessed at hospital admission, with definitions based on previous studies [35, 36]. Diabetes mellitus was defined based on a previous diagnosis, use of antidiabetic medication, or elevated blood glucose according to clinical records. Hypertension was defined based on a previous diagnosis, antihypertensive medication use, or elevated blood pressure documented during clinical evaluation. Kidney disease was identified from medical history or abnormal renal function markers. Family history of coronary artery disease was recorded when a first-degree relative had a documented history of coronary artery disease. Smoking status was defined as current or previous tobacco use. The total number of risk factors was then counted for each patient and categorized as one, two, or three or more AMI risk factors to reflect increasing risk-factor burden.

### Laboratory analysis

Venous blood was obtained from patients with AMI at hospital admission. For AMI patients, venous blood sampling was performed within 2 h after hospital admission, and the reported symptom onset-to-admission interval ranged from 3 to 48 h before hospital arrival. For healthy controls, sampling was performed in the morning after overnight fasting. Standard laboratory measurements included total cholesterol (TC), LDL-c, HDL-c, triglycerides (TG), random blood sugar (RBS), urea, and creatinine, all analyzed in the hospital laboratory.

Adiponectin, GDF15, and IL-6 were quantified using the Luminex^®^ Discovery Assay Human Premixed Multi-analyte Kit (LXSAHM-05; lot L151823). Serum FGF21 was measured with a Quantikine^®^ Human FGF21 ELISA kit (R&D Systems, Minneapolis, MN, USA; lot P128541), and absorbance was read on a Model 680 microplate reader (Bio-Rad Laboratories, Hercules, CA, USA).

### Statistical analysis

Data were analyzed in GraphPad Prism version 10.0 (GraphPad Software, LLC, San Diego, CA, USA). Continuous variables are reported as mean ± standard deviation (SD) when normally distributed and as median (minimum–maximum) when non-normally distributed. Data distribution was assessed using the Shapiro–Wilk test. Categorical variables are expressed as frequency and percentage. Categorical variables are expressed as frequency and percentage. Between-group comparisons used independent-sample t-tests or Mann-Whitney U tests, as appropriate. Relationships between adipokines and lipid parameters were assessed using Pearson or Spearman correlation coefficients according to data distribution. Additional exploratory regression analyses were conducted as supplementary analyses to examine crude and covariate-adjusted associations between circulating adipokines and selected clinical, anthropometric, and metabolic variables. The adjusted models included age, sex, AMI type, AMI risk-factor burden, lipid parameters, insulin, creatinine, and adiposity indices, as appropriate. A two-sided p value < 0.05 was considered statistically significant.

### Ethical considerations

This study was approved by the Ethics Committee of Universitas Padjadjaran, Indonesia (Approval No. 094/UN6.KEP/EC/2022) and was conducted in accordance with the Declaration of Helsinki. Written informed consent was obtained from all participants.

## Results

A total of 105 AMI patients were included in the final analysis, comprising 62 non-obese individuals (BMI < 25 kg/m²) and 43 obese individuals (BMI ≥ 25 kg/m²). The majority of patients in both groups were male (66.1% in the non-obese group and 86.1% in the obese group), and most were over 50 years of age.

Obese patients had significantly higher values for weight, height, body fat percentage, waist circumference (WC), and waist-to-height ratio (WHtR) compared to their non-obese counterparts (all *p* < 0.001). No significant differences were observed between the groups in terms of systolic and diastolic blood pressure, age, or cardiac troponin I levels.

Regarding lipid profiles, total cholesterol (*p* = 0.0041), LDL cholesterol (*p* = 0.0034), and triglyceride levels (*p* = 0.0023) were significantly higher in the obese group. HDL cholesterol levels were lower in the obese group, but the difference was not statistically significant (*p* = 0.1077). Insulin levels were significantly elevated in the obese group (*p* = 0.0146), while glucose, urea, and creatinine levels were comparable between the two groups (see Table 1).

**Table 1:** Baseline Characteristics of Patients with AMI (*n* = 105)

Variable		Non-obese(*n* = 62)	Obese (43)	*p* value
Demographic and Clinical Characteristics				
Gender, *n* (%)				
	Male	41 (66.13)	37 (86.05)	
	Female	21 (33.87)	6 (13.95)	
AMI, *n* (%)				
	STEMI	29 (46.77)	31 (72.09)	
	NSTEMI	33 (53.23)	12 (27.91)	
AMI + Risk Factor, *n* (%)				
	1 risk factor	27 (43.55)	10 (23.26)	
	2 risk factors	27 (43.55)	23 (53.48)	
	≥ 3 risk factors	8 (12.90)	10 (23.26)	
Age (years), Mean ± SD		59 ± 11.07	54.79 ± 11.15	0.0588
Body Anthropometry				
Systolic Blood Pressure (mmHg), Mean ± SD		122.4 ± 18.21	126.8 ± 26.13	0.3058
Diastolic Blood Pressure (mmHg), Mean ± SD		74.94 ± 11.75	78.02 ± 13.34	0.2132
Body fat, Mean ± SD		19.69 ± 8.146	28.98 ± 6.568	<0.0001
Waist Circumference, Mean ± SD		87.65 ± 7.618	99.07 ± 5.557	0.0078
WHtR, Mean ± SD		0.546 ± 0.052	0.594 ± 0.038	< 0.0001*
Laboratory Parameters				
Cardiac Troponin I (ng/mL), Median (min-max)		1.57 (0.02-10)	0.88 (0.02-10)	0.7461
WBC (mm³), Median (min-max)		11,475 (5660–21720)	10,600 (7560–22640)	0.5242
TC (mg/dL), Median (min-max)		148 (69–359)	178 (104–254)	0.0041*
HDL-c (mg/dL), Mean ± SD		35.56 ± 8.121	33.19 ± 6.165	0.1077
LDL-c (mg/dL), Mean ± SD		106.8 ± 35.7	127.6 ± 33.76	0.0034*
TG (mg/dL), Median (min-max)		108 (46–455)	138 (37–336)	0.0023*
RBS (mg/dL), Median (min-max)		125 (75–548)	123 (78–310)	0.9677
Insulin (pg/mL), Median (min-max)		437.2 (253.3–3090)	585.2 (280.1–4047)	0.0146*
Urea (mg/dL), Median (min-max)		30.75 (14.3-204.4)	26.2 (5.7-153.2)	0.1653
Creatinine (mg/dL), Median (min-max)		1.095 (0.59–22.8)	1.06 (0.41–23.5)	0.431

Data are presented as mean ± SD, median (min–max), or frequencies (%). Statistical tests used include independent t-tests and Mann–Whitney U tests depending on data distribution. *Significance set at *p* < 0.05

*Abbreviations* *SD * standard deviation, *AMI * acute myocardial infarction, *STEMI * ST-elevation myocardial infarction, *NSTEMI * non-ST-elevation myocardial infarction, *WHtR * waist-to-height ratio, *WBC * white blood cell, *TC * total cholesterol, *HDL-c * high-density lipoprotein cholesterol, *LDL-c * low-density lipoprotein cholesterol, *TG * triglycerides, *RBS * random blood sugar

**Fig. 2:**
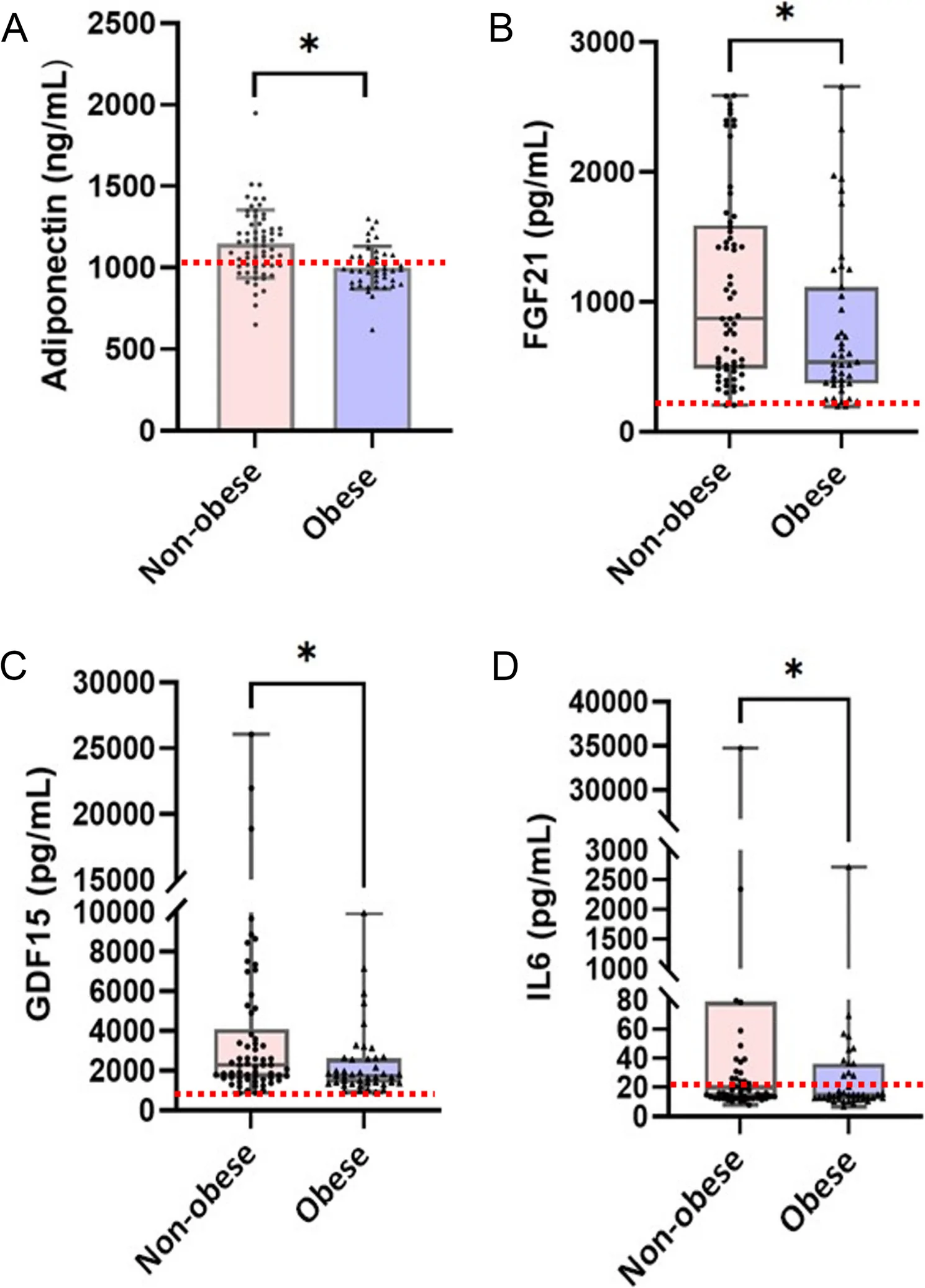
Circulating adipokine and inflammatory cytokine levels in patients with acute myocardial infarction according to obesity status. Circulating levels of (**A**) adiponectin, (**B**) FGF21, (**C**) GDF15, and (**D**) IL-6 were compared between non-obese and obese patients with AMI. The dashed red line represents the reference value derived from 10 healthy individuals sampled under fasting conditions. This reference line is presented for descriptive visual context only and was not used as a formal comparator in statistical hypothesis testing. Numerical values for each biomarker are provided in Supplementary Table S9. A p value < 0.05 was considered statistically significant

As shown in Fig. 2, serum levels of adiponectin, FGF21, GDF15, and IL-6 were significantly higher in AMI patients classified as non-obese than in obese patients (all *p* < 0.05). In crude regression analysis, BMI was associated with adiponectin; however, this association was no longer significant after adjustment for demographic, clinical, metabolic, and renal-function variables. BMI was also not independently associated with FGF21, GDF15, or IL-6, suggesting that adipokine differences by obesity status may partly reflect clinical and metabolic confounding, as shown in Supplementary Tables S1 to S8.

Adiponectin levels were significantly higher in non-obese patients than in obese patients and were comparable to the healthy control reference (Fig. 2A; *p* < 0.05). In contrast, FGF21 concentrations exceeded the healthy control reference in both groups, with significantly higher levels observed in non-obese patients (Fig. 2B; *p* < 0.05). Similarly, GDF15 levels were markedly elevated above the healthy control reference in both groups, with higher concentrations in non-obese patients (Fig. 2C; *p* < 0.05). IL-6 levels also exceeded the healthy control reference in both groups and were significantly higher in non-obese patients compared with obese patients (Fig. 2D; *p* < 0.05). Numerical values for each biomarker are provided in Supplementary Table S9.

To further evaluate whether BMI was associated with circulating adipokine levels, crude and covariate-adjusted regression analyses were performed (Table 2). BMI was significantly associated with higher adiponectin levels in the crude model (β = 97.94; 95% CI: 23.76 to 172.1; *p* = 0.0102), but this association was no longer significant after adjustment for demographic, clinical, lipid, metabolic, and renal variables (β = 40.97; 95% CI: −69.95 to 151.9; *p* = 0.4651). BMI was not significantly associated with FGF21, GDF15, or IL-6 in either crude or adjusted analyses. Although the IL-6 estimate changed direction after adjustment, the association remained non-significant with wide confidence intervals.

**Table 2:** Crude and adjusted associations between BMI and circulating adipokine levels in patients with acute myocardial infarction

Outcome	Crude*			Adjusted**		
	Estimate	95% CI	*p*-value	Estimate	95% CI	*p*-value
Adiponectin	97.94	23.76 to 172.1	0.0102	40.97	-69.95 to 151.9	0.4651
FGF21	148.2	-130.6 to 427.0	0.2942	264.4	-163.9 to 692.6	0.2234
GDF15	1003	-532.0 to 2539	0.1978	55.42	-2264 to 2375	0.9623
IL-6	827.9	-649.1 to 2305	0.2689	-869.8	-3195 to 1455	0.4595

* Crude model included BMI only. **Adjusted model included age, sex, AMI risk-factor burden, AMI type, LDL-c, HDL-c, triglycerides, insulin, and creatinine. β = regression coefficient; CI = confidence interval. The direction of the IL-6 estimate changed after adjustment, but the association remained statistically non-significant

Table 3 shows the correlations between circulating adipokines and lipid parameters in all AMI patients and according to obesity status. Among all patients, adiponectin was negatively correlated with total cholesterol and triglycerides after FDR correction. GDF15 was negatively correlated with total cholesterol and LDL-c, while IL-6 was negatively correlated with total cholesterol and triglycerides. FGF21 showed no significant correlation with lipid parameters after FDR correction. In subgroup analyses, no adipokine–lipid correlations remained significant after FDR correction in either non-obese or obese patients.

**Table 3:** Correlations Between Adipokine Levels and Lipid Parameters in AMI Patients

	All patients (*n* = 105)				Non-obese (*n* = 62)			Obese (*n* = 43)				
	*r*	*p*	Cl	*p* FDR	*r*	*p*	Cl	*p* FDR	*r*	*p*	Cl	*p* FDR
Adiponectin												
TC (mg/dL)	-0.242*	0.013	(-0.419, -0.047)	0.037	-0.086	0.505	(-0.336, 0.175)	0.621	-0.211	0.173	(-0.488, 0.104)	0.542
HDL-c (mg/dL)	0.214*	0.028	(0.18, 0.395)	0.056	0.203	0.113	(-0.056, 0.437)	0.361	0.213	0.170	(-0.102, 0.490)	0.542
LDL-c (mg/dL)	-0.215*	0.028	(-0.395, 0.018)	0.056	-0.071	0.584	(-0.322, 0.189)	0.667	-0.284	0.065	(-0.545, 0.027)	0.542
TG (mg/dL)	-0.287**	0.003	(-0.458, 0.095)	0.032	-0.234	0.068	(-0.463, 0.025)	0.272	-0.148	0.342	(-0.437, 0.168)	0.608
FGF21												
TC (mg/dL)	-0.116	0.238	(-0.306, 0.083)	0.292	0.056	0.664	(-0.203, 0.309)	0.68	-0.172	0.271	(-0.456, 0.145)	0.542
HDL-c (mg/dL)	-0.040	0.683	(-0.236, 0.158)	0.683	-0.153	0.235	(-0.394, 0.108)	0.456	-0.017	0.912	(-0.293, 0.324)	0.933
LDL-c (mg/dL)	-0.042	0.670	(-0.237, 0.156)	0.683	0.100	0.440	(-0.161, 0.348)	0.621	-0.037	0.814	(-0.342, 0.275)	0.930
TG (mg/dL)	-0.203*	0.038	(-0.385, 0.006)	0.060	-0.129	0.316	(-0.374, 0.132)	0.505	-0.133	0.396	(-0.424, 0.183)	0.633
GDF15												
TC (mg/dL)	-0.252**	0.010	(-0.428, -0.057)	0.037	-0.146	0.257	(-0.388, 0.115)	0.456	-0.281	0.068	(-0.542, 0.031)	0.542
HDL-c (mg/dL)	-0.206*	0.035	(-0.387, 0.009)	0.060	-0.289*	0.023	(-0.508, -0.034)	0.184	-0.175	0.261	(-0.459, 0.141)	0.542
LDL-c (mg/dL)	-0.240*	0.014	(-0.417, -0.045)	0.037	-0.148	0.251	(-0.390, 0.113)	0.456	-0.181	0.245	(-0.464, 0.135)	0.542
TG (mg/dL)	-0.134	0.172	(-0.323, 0.065)	0.229	-0.053	0.680	(-0.306, 0.206)	0.68	-0.063	0.687	(-0.365, 0.250)	0.857
IL-6												
TC (mg/dL)	-0.257**	0.008	(-0.433, -0.063)	0.037	-0.240	0.060	(-0.468, 0.018)	0.272	-0.214	0.168	(-0.490, 0.101)	0.542
HDL-c (mg/dL)	-0.061	0.533	(-0.137, 0,256)	0.609	0.088	0.496	(-0.173, 0.337)	0.621	-0.072	0.649	(-0.372, 0.242)	0.857
LDL-c (mg/dL)	-0.179	0.068	(-0.363, 0.019)	0.098	-0.176	0.171	(-0.414, 0.085)	0.456	-0.061	0.697	(-0.363, 0.252)	0.857
TG (mg/dL)	-0.278**	0.004	(-0.450, -0.085)	0.032	-0.363**	0.004	(-0.567, -0.117)	0.064	-0.013	0.933	(-0.321, 0.297)	0.933

The correlation coefficients (*r*) and *p*-values for the association between adipokine concentrations and lipid profile parameters (TC, HDL-c, LDL-c, and TG) in all patients, AMI patients with non-obese, and obese subgroups

*Abbreviations* *TC * total cholesterol, *HDL-c * high-density lipoprotein cholesterol, *LDL-c * low-density lipoprotein cholesterol, *TG * triglycerides, *FDR * false discovery rate

Crude regression analyses further showed that several adiposity indicators were associated with circulating adipokine levels (Supplementary Tables S1–S8). In the crude models, BMI was positively associated with adiponectin, while waist circumference, body fat percentage, and waist-to-height ratio showed inverse associations with adiponectin. Waist circumference was also inversely associated with FGF21, GDF15, and IL-6, whereas waist-to-height ratio was inversely associated with IL-6. However, after adjustment for demographic, clinical, lipid, metabolic, renal, and adiposity-related variables, most associations between adiposity indicators and adipokines were attenuated and no longer statistically significant. This pattern suggests that the observed adipokine profiles may be influenced by multiple interrelated clinical and metabolic factors rather than by adiposity indicators alone.

## Discussion

Obesity is widely recognized as a major risk factor for cardiometabolic diseases, including AMI, and thus anthropometric status is often presumed to influence adipokine secretion and AMI pathogenesis. In this study, circulating adipokine and inflammatory cytokine levels were higher in non-obese patients during the early phase of AMI compared with obese patients.

Adiponectin, FGF21, and GDF15, three adipokines associated with anti-inflammatory, metabolic-regulatory, and cardioprotective functions, showed higher circulating concentrations in the non-obese group. However, given the cross-sectional design and single time-point measurement, these findings should be interpreted as associative differences according to obesity status rather than direct evidence of a preserved adaptive response to acute ischemic stress. Conversely, obese patients showed lower circulating adipokine levels, although this finding should be interpreted cautiously because of the cross-sectional design and potential residual confounding.

Meanwhile, IL-6 is a pleiotropic mediator that exerts both proinflammatory and anti-inflammatory effects, was also markedly increased in non-obese patients, further highlighting the dynamic activation of adipose-derived signaling during the acute phase of AMI in those without excessive adiposity. These observations indicate BMI-related differences in circulating inflammatory and metabolic biomarker profiles during AMI, but do not establish preserved compensatory mechanisms.

Among the well-characterized adipokines, adiponectin has been widely investigated in relation to obesity, cardiometabolic risk, and AMI, although findings across populations and clinical settings have not been entirely consistent. Previous clinical studies have reported altered circulating adiponectin levels in patients with AMI, including non-obese populations, although findings across populations and study designs remain variable [25].

Obesity further modifies this relationship, with previous clinical evidence indicating that obese patients with AMI may have lower circulating adiponectin levels than non-obese patients with AMI [27]. Our findings are consistent with this evidence, reinforcing the inverse association between adiponectin and BMI as well as other indicators of adiposity. Interestingly, our study applied a different operational definition of obesity compared with previous studies, largely reflecting differences in genetic and ethnic backgrounds (Asian versus Caucasian populations). This highlights the importance of considering ancestry-specific adiposity thresholds when defining obesity in both clinical practice and research, particularly in cardiometabolic risk evaluation.

Because the healthy control values were included only as descriptive visual references and were not used for formal statistical comparisons, interpretation of Fig. 2 should focus on differences between non-obese and obese AMI patients. The reference line provides contextual orientation only and should not be interpreted as evidence of significant differences between AMI patients and healthy individuals.

Previous studies have investigated circulating adiponectin levels in patients with AMI, including non-obese populations, although findings across clinical settings remain variable 25]. In the present study, adiponectin levels were higher in non-obese than in obese AMI patients. Previous experimental studies have suggested that adiponectin may be involved in pathways related to myocardial perfusion, oxidative stress, and ischemic injury [22, 37, 38]. However, these mechanisms were not directly assessed in the present study and should be interpreted only as biological context. Clinically, higher adiponectin has been associated with myocardial salvage, recovery of left ventricular function, and lower risk of adverse cardiovascular events after AMI [26, 39–41].

At the same time, adiponectin biology is not unidirectional. Higher circulating levels have also been associated with increased long-term mortality in AMI and coronary artery disease cohorts, an apparent paradox that may reflect reverse causation, greater illness severity, or the influence of comorbidity burden on adiponectin regulation [42–44].

FGF21 and GDF15 also showed higher circulating levels in non-obese AMI patients than in obese patients, suggesting BMI-related differences in stress-associated biomarker profiles. In prior AMI studies, circulating FGF21 rises sharply soon after symptom onset and can remain elevated for several days, consistent with a stress-inducible endocrine program activated by myocardial ischemia [45]. These observations suggest a sustained stress-responsive secretion pattern during acute myocardial injury. Our data provide additional insight by showing that FGF21 levels were higher in non-obese AMI patients compared with obese AMI patients. This finding is intriguing because, under baseline (non-ischemic) conditions, serum FGF21 levels are typically higher in obese individuals than in healthy non-obese [46].

Beyond obesity, FGF21 increases in several critical illnesses and is increasingly viewed as a systemic stress hormone [29]. Within the heart, FGF21 plays an essential protective role in ventricular remodeling. Experimental studies demonstrated that FGF21 administration in a murine MI model attenuated myocardial inflammation evidenced by reduced IL-6 and TNF-α expression, improved cardiac function and limited pathological remodeling, partly through adiponectin-dependent pathways [47].

GDF15 and FGF21 exhibited a similar obesity-related pattern in the context of AMI. In our study, GDF15 levels were higher in non-obese AMI patients than in those with obesity, a trend that opposes the baseline influence of adiposity. Under non-ischemic conditions, obesity is associated with significantly elevated circulating GDF15 levels, and these levels correlate positively with adiposity parameters [48].

Prior human studies have linked GDF15 to abdominal obesity, insulin resistance, and future deterioration of glucose homeostasis [49]. Expression is enriched in visceral adipose tissue and rises with adipose macrophage infiltration and hepatocyte stress during progression from obesity to metabolic liver disease, emphasizing its connection to systemic metabolic stress rather than to adiposity alone [50].

Similar to FGF21, GDF15 secretion is induced by cellular and metabolic stress^51^, including mitochondrial dysfunction, inflammation, and ischemic injury [51–54]. Experimental studies have suggested that GDF15 may be involved in ischemia–reperfusion injury and apoptosis-related pathways. However, the present study did not assess myocardial tissue expression, downstream signaling, or functional myocardial responses. In vitro studies further support that GDF15 is actively secreted by cardiomyocytes exposed to simulated ischemia/reperfusion, indicating possible autocrine and paracrine cardioprotective actions [55].

The higher GDF15 levels observed in non-obese AMI patients may reflect differences in circulating stress-associated biomarker profiles; however, the present study cannot determine whether this represents a protective response. Previous experimental studies suggest that FGF21 may modulate inflammation and post-infarction remodeling, whereas GDF15 may be involved in ischemia–reperfusion injury and apoptosis-related pathways; however, these mechanisms were not directly assessed in the present study [55–57].

However, IL-6 is a pleiotropic cytokine with multiple cellular sources. In addition to adipose tissue, IL-6 can also be secreted by immune cells, endothelial cells, cardiomyocytes, skeletal muscle, and other tissues, particularly during systemic inflammation and acute cardiovascular injury. Nevertheless, IL-6 has frequently been discussed in the literature as an adipose tissue-derived inflammatory mediator and is commonly included within the broader adipokine/adipocytokine framework, especially in studies addressing obesity, metabolic inflammation, and cardiovascular disease [58–60].

In the context of AMI, IL-6 plays an important role in the dynamic and highly coordinated innate immune response after myocardial infarction. Other members of the IL-6 cytokine family, such as oncostatin M, have also been implicated in cardiovascular inflammation, angiogenesis, remodeling, and tissue injury, supporting the concept that IL-6-family cytokine signaling may have context-dependent roles during myocardial injury [61]. In general, IL-6 rises during the acute phase of AMI in response to myocardial necrosis/ischemia, endothelial activation, and leukocyte recruitment, and it contributes to the hepatic acute-phase response. These IL-6 surges have been associated with systemic inflammatory burden and the severity of myocardial injury, including associations with infarct size and adverse outcomes in STEMI cohorts [62, 63].

Obesity may reshape this response because adipose tissue itself is a chronic source of IL-6 and other cytokines, thereby establishing an altered inflammatory set point before the infarction occurs [64, 65]. Consistent with this framework, our study indicates that Circulating IL-6 levels differed according to BMI status, with lower levels observed in obese than in non-obese patients in this cross-sectional analysis.

In the present cross-sectional analysis, circulating IL-6 levels tended to be lower in obese compared with non-obese patients. Although this finding should be interpreted cautiously, it may reflect altered inflammatory regulation associated with obesity. Obesity is characterized by chronic low-grade inflammation and dysregulated cytokine signaling, which can modify the systemic inflammatory profile and the balance of pro- and anti-inflammatory mediators. Consequently, rather than sustaining a coordinated inflammatory response that facilitates debris clearance and tissue repair, individuals with obesity may exhibit a relatively blunted or dysregulated cytokine response in acute cardiovascular injury. However, given the cross-sectional design of the present study, these interpretations remain speculative and require confirmation in longitudinal studies examining temporal cytokine dynamics [66].

IL-6 is secreted by multiple cell types, including macrophages, adipocytes, endothelial cells, and cardiomyocytes. Its biological effects are context-dependent, but receptor activity and downstream signaling pathways were not evaluated in this study; these pathways can be context-dependent, supporting acute-phase responses and healing when appropriately regulated, but contributing to inflammatory amplification and maladaptive remodeling when excessive or persistent [67]. Within this framework, the lower circulating IL-6 levels observed in obese patients may reflect altered inflammatory regulation; however, this interpretation remains speculative and requires longitudinal validation [64, 65].

Our study demonstrated lower total TC and LDL-c levels, with no significant difference in HDL-c, among non-obese patients presenting with AMI. These findings are consistent with previous reports, lipid profiles during AMI are known to vary considerably, influenced by the acute-phase response as well as patient demographics and underlying metabolic status.

In this study, FGF21 showed an inverse correlation with HDL-c in non-obese AMI patients, while GDF15 was inversely associated with TC and LDL-c levels. Moreover, HDL-c levels showed an inverse association with high-sensitivity C-reactive protein (hs-CRP), suggesting that reduced HDL-c concentrations may be related to greater systemic inflammatory activity and poorer clinical outcomes [68]. These observations raise the possibility that elevated FGF21 levels in non-obese individuals may play a protective metabolic or cardiovascular role, although this hypothesis warrants further investigation. Higher LDL-c levels during the acute phase of AMI have generally been associated with worse in-hospital outcomes. Nevertheless, apparently discordant findings have also been described, where higher admission LDL-c levels are linked to better outcomes in certain subgroups, particularly among patients with STEMI [69]. In this context, the role of GDF15 in relation to LDL-c dynamics during AMI remains unclear and deserves more detailed exploration, especially in AMI patients with obesity.

Interpretation of these results should take into account the dynamic nature of lipid profiles during the acute phase of AMI, which may include transient reductions in LDL-c, total cholesterol, and HDL-c, along with relatively stable or elevated triglyceride levels due to systemic inflammatory responses [70, 71]. Therefore, associations between adipokines and lipid parameters during this phase should be interpreted cautiously and in the context of patients’ baseline metabolic status.

An important unresolved question is how dynamic changes in circulating adipokines influence clinical outcomes and prognosis following AMI across different BMI categories. The observed increases in adiponectin, FGF21, GDF15, and IL-6 may represent a multifaceted response involving acute systemic inflammation triggered by coronary artery occlusion, local signaling within the injured myocardium and affected vascular tissues, and pre-existing metabolic or inflammatory states that preceded the infarction. Rather than reflecting a single protective or harmful pathway, these biomarker alterations may indicate heterogeneous circulating inflammatory and metabolic responses to acute cardiovascular stress. Longitudinal follow-up after the acute phase will be essential to determine whether specific adipokine trajectories are associated with myocardial recovery, adverse remodeling, recurrent events, or long-term cardiovascular risk. Such studies may also help clarify the biological underpinnings of the so-called counterintuitive BMI-outcome pattern in AMI [72].

Finally, adipose tissue should not be viewed as the sole source of the molecules measured in this study. The myocardium, vascular endothelium, liver, and immune system may all contribute to the circulating signal. Future work combining serial sampling with tissue-specific mechanistic studies will be needed to disentangle source, timing, and function of these mediators after AMI.

## Conclusion

This study found that non-obese AMI patients had higher circulating levels of adiponectin, FGF21, GDF15, and IL-6 than obese patients. These differences indicate BMI-related variation in circulating inflammatory and metabolic biomarker profiles during AMI and should not be interpreted as evidence of a preserved adaptive response. Adiponectin, GDF15, and IL-6 showed inverse associations with selected lipid parameters, particularly in all patients. Overall, these findings highlight associations between adiposity status and circulating biomarker patterns during the acute phase of AMI. Given the cross-sectional design, causal relationships and prognostic implications cannot be established, and further longitudinal studies are required to clarify the clinical relevance of these observations.

### Clinical implications

Non-obese patients with AMI exhibited higher circulating levels of adiponectin, FGF21, GDF15, and IL-6 compared with obese patients, despite comparable levels of myocardial injury. These observations indicate that adiposity status is associated with differences in circulating inflammatory and metabolic biomarker profiles during AMI. The distinct circulating inflammatory and metabolic biomarker profiles observed highlight the potential value of adipokine profiling for characterizing BMI‑related heterogeneity in AMI. Further studies are needed to evaluate the prognostic value and clinical relevance of these biomarker patterns in AMI.

### Limitations

This study has several limitations. Its cross-sectional design does not permit conclusions about causal relationships between adipokine levels, obesity status, and myocardial injury. Biomarkers were measured at a single time point during the acute phase of AMI; therefore, temporal changes in adipokines and lipid parameters could not be assessed. The sample size may not fully capture within-group variability related to fat distribution, and no formal a priori power calculation was performed; therefore, subgroup analyses should be interpreted as exploratory. Although BMI, waist circumference, body fat percentage, and waist-to-height ratio were assessed, this study did not directly measure visceral adipose tissue or epicardial adipose tissue. Thus, obesity should be interpreted as clinically observable anthropometric adiposity rather than a direct measure of metabolically active visceral or cardiac fat depots. Circulating biomarker levels were assessed without evaluating local tissue expression, receptor activity, downstream signaling, or functional myocardial assays. Although supplementary crude and adjusted regression analyses were performed, residual confounding remains possible because factors such as insulin resistance, lifestyle behaviors, medication exposure, nutritional status, and inflammatory comorbidities were not fully captured. Finally, the absence of follow-up data limits interpretation of prognostic implications. Future studies should incorporate serial biomarker measurements and imaging-based assessment of visceral and epicardial fat to better characterize adiposity-related adipokine patterns in AMI.

## Supplementary Information


Supplementary Material 1.


## Data Availability

The de-identified datasets generated and/or analyzed during the current study are available from the corresponding author on reasonable request. The data are not publicly available due to privacy and ethical restrictions related to clinical patient information.

## Abbreviations

AMI: Acute myocardial infarction
BF: Body fat
BIA: Bioelectrical impedance analysis
BMI: Body mass index
CI: Confidence interval
CVD: Cardiovascular disease
ECG: Electrocardiogram
ELISA: Enzyme-linked immunosorbent assay
FDR: False discovery rate
FGF21: Fibroblast growth factor 21
GDF15: Growth differentiation factor 15
HDL-c: High-density lipoprotein cholesterol
hs-CRP: High-sensitivity C-reactive protein
IL-6: Interleukin-6
IL-6R: Interleukin-6 receptor
IQR: Interquartile range
LDL-c: Low-density lipoprotein cholesterol
NSTEMI: Non-ST-segment elevation myocardial infarction
RBS: Random blood sugar
SD: Standard deviation
STEMI: ST-segment elevation myocardial infarction
TC: Total cholesterol
TG: Triglycerides
WC: Waist circumference
WBC: White blood cell
WHtR: Waist-to-height ratio
