# Supplementary material for: Adipokine-mediated metabolic responses across BMI in acute myocardial infarction
Source: BMC Cardiovasc Disord. 2026 Jun 20;26:763. doi: 10.1186/s12872-026-06140-w (PMC13531907; doi:10.1186/s12872-026-06140-w)
Supplement: Supplementary file 1 [file 12872_2026_6140_MOESM1_ESM.docx]

**SUPPLEMENTARY**

| **Supplementary Table S.1.1 Crude regression models for adiponectin**  **(Body Mass Index)** | | | | | | | |
| --- | --- | --- | --- | --- | --- | --- | --- |
| **Parameter estimates** | **Variable** | **Estimate** | **Standard error** | **95% CI (asymptotic)** | **\|t\|** | **P value** | **P value summary** |
| β0 | Intercept | 1029 | 28.75 | 972.2 to 1086 | 35.81 | <0.0001 | **** |
| β1 | BMI | 97.94 | 37.41 | 23.76 to 172.1 | 2.618 | 0.0102 | * |

| **Supplementary Table S.1.2 Crude regression models for FGF21**  **(Body Mass Index)** | | | | | | | |
| --- | --- | --- | --- | --- | --- | --- | --- |
|  | | | | | | | |
| **Parameter estimates** | **Variable** | **Estimate** | **Standard error** | **95% CI (asymptotic)** | **\|t\|** | **P value** | **P value summary** |
| β0 | Intercept | 896.5 | 108 | 682.2 to 1111 | 8.299 | <0.0001 | **** |
| β1 | BMI | 148.2 | 140.6 | -130.6 to 427.0 | 1.054 | 0.2942 | ns |

| **Supplementary Table S.1.3 Crude regression models for GDF15**  **(Body Mass Index)** | | | | | | | |
| --- | --- | --- | --- | --- | --- | --- | --- |
| **Parameter estimates** | **Variable** | **Estimate** | **Standard error** | **95% CI (asymptotic)** | **\|t\|** | **P value** | **P value summary** |
| β0 | Intercept | 2726 | 594.9 | 1546 to 3906 | 4.582 | <0.0001 | **** |
| β1 | BMI | 1003 | 774.2 | -532.0 to 2539 | 1.296 | 0.1978 | ns |

| **Supplementary Table S.1.4 Crude regression models for IL-6**  **(Body Mass Index)** | | | | | | | |
| --- | --- | --- | --- | --- | --- | --- | --- |
| **Parameter estimates** | **Variable** | **Estimate** | **Standard error** | **95% CI (asymptotic)** | **\|t\|** | **P value** | **P value summary** |
| β0 | Intercept | 302.1 | 572.3 | -832.8 to 1437 | 0.528 | 0.5987 | ns |
| β1 | BMI | 827.9 | 744.7 | -649.1 to 2305 | 1.112 | 0.2689 | ns |

| **Supplementary Table S.2.1 Crude regression models for adiponectin**  **(Waist Circumference)** | | | | | | | |
| --- | --- | --- | --- | --- | --- | --- | --- |
| **Parameter estimates** | **Variable** | **Estimate** | **Standard error** | **95% CI (asymptotic)** | **\|t\|** | **P value** | **P value summary** |
| β0 | Intercept | 1789 | 187.5 | 1417 to 2161 | 9.538 | <0.0001 | **** |
| β1 | Waist Circumference | -7.599 | 2.022 | -11.61 to -3.589 | 3.758 | 0.0003 | *** |
| **Supplementary Table S.2.2 Crude regression models for FGF21**  **(Waist Circumference)** | | | | | | | |
| **Parameter estimates** | **Variable** | **Estimate** | **Standard error** | **95% CI (asymptotic)** | **\|t\|** | **P value** | **P value summary** |
| β0 | Intercept | 2436 | 717.3 | 1013 to 3858 | 3.396 | 0.0010 | *** |
| β1 | Waist Circumference | -15.72 | 7.734 | -31.06 to -0.3852 | 2.033 | 0.0446 | * |
| **Supplementary Table S.2.3 Crude regression models for GDF15**  **(Waist Circumference)** | | | | | | | |
| **Parameter estimates** | **Variable** | **Estimate** | **Standard error** | **95% CI (asymptotic)** | **\|t\|** | **P value** | **P value summary** |
| β0 | Intercept | 12318 | 3941 | 4503 to 20133 | 3.126 | 0.0023 | ** |
| β1 | Waist Circumference | -97.48 | 42.49 | -181.8 to -13.22 | 2.294 | 0.0238 | * |
| **Supplementary Table S.2.4 Crude regression models for IL-6**  **(Waist Circumference)** | | | | | | | |
| **Parameter estimates** | **Variable** | **Estimate** | **Standard error** | **95% CI (asymptotic)** | **\|t\|** | **P value** | **P value summary** |
| β0 | Intercept | 9180 | 3788 | 1668 to 16693 | 2.424 | 0.0171 | * |
| β1 | Waist Circumference | -90.87 | 40.84 | -171.9 to -9.864 | 2.225 | 0.0283 | * |

| **Supplementary Table S.3.1 Crude regression models for adiponectin**  **(Body Fat)** | | | | | | | |
| --- | --- | --- | --- | --- | --- | --- | --- |
| **Parameter estimates** | **Variable** | **Estimate** | **Standard error** | **95% CI (asymptotic)** | **\|t\|** | **P value** | **P value summary** |
| β0 | Intercept | 1246 | 51.77 | 1143 to 1348 | 24.06 | <0.0001 | **** |
| β1 | Body Fat | -6.744 | 2.065 | -10.84 to -2.649 | 3.266 | 0.0015 | ** |

| **Supplementary Table S.3.2 Crude regression models for FGF21**  **(Body Fat)** | | | | | | | |
| --- | --- | --- | --- | --- | --- | --- | --- |
| **Parameter estimates** | **Variable** | **Estimate** | **Standard error** | **95% CI (asymptotic)** | **\|t\|** | **P value** | **P value summary** |
| β0 | Intercept | 1164 | 198.1 | 771.0 to 1557 | 5.876 | <0.0001 | **** |
| β1 | Body Fat | -7.654 | 7.899 | -23.32 to 8.013 | 0.9689 | 0.3348 | ns |

| **Supplementary Table S.3.3 Crude regression models for GDF15**  **(Body Fat)** | | | | | | | |
| --- | --- | --- | --- | --- | --- | --- | --- |
| **Parameter estimates** | **Variable** | **Estimate** | **Standard error** | **95% CI (asymptotic)** | **\|t\|** | **P value** | **P value summary** |
| β0 | Intercept | 5320 | 1078 | 3181 to 7459 | 4.933 | <0.0001 | **** |
| β1 | Body Fat | -85.21 | 43.01 | -170.5 to 0.08760 | 1.981 | 0.0502 | ns |

| **Supplementary Table S.3.4 Crude regression models for IL-6**  **(Body Fat)** | | | | | | | |
| --- | --- | --- | --- | --- | --- | --- | --- |
| **Parameter estimates** | **Variable** | **Estimate** | **Standard error** | **95% CI (asymptotic)** | **\|t\|** | **P value** | **P value summary** |
| β0 | Intercept | 2400 | 1041 | 335.4 to 4465 | 2.305 | 0.0231 | * |
| β1 | Body Fat | -68.49 | 41.52 | -150.8 to 13.85 | 1.65 | 0.1021 | ns |

| **Supplementary Table S.4.1 Crude regression models for adiponectin**  **(Waist to Height Ratio)** | | | | | | | |
| --- | --- | --- | --- | --- | --- | --- | --- |
| **Parameter estimates** | **Variable** | **Estimate** | **Standard error** | **95% CI (asymptotic)** | **\|t\|** | **P value** | **P value summary** |
| β0 | Intercept | 1621 | 199.3 | 1226 to 2017 | 8.136 | <0.0001 | **** |
| β1 | Waist to Height Ratio | -944.4 | 350.7 | -1640 to -248.8 | 2.693 | 0.0083 | ** |

| **Supplementary Table S.4.2 Crude regression models for FGF21**  **(Waist to Height Ratio)** | | | | | | | |
| --- | --- | --- | --- | --- | --- | --- | --- |
| **Parameter estimates** | **Variable** | **Estimate** | **Standard error** | **95% CI (asymptotic)** | **\|t\|** | **P value** | **P value summary** |
| β0 | Intercept | 1045 | 754.2 | -450.4 to 2541 | 1.386 | 0.1687 | ns |
| β1 | Waist to Height Ratio | -108.5 | 1327 | -2741 to 2524 | 0.08171 | 0.935 | ns |

| **Supplementary Table S.4.3 Crude regression models for GDF15**  **(Waist to Height Ratio)** | | | | | | | |
| --- | --- | --- | --- | --- | --- | --- | --- |
| **Parameter estimates** | **Variable** | **Estimate** | **Standard error** | **95% CI (asymptotic)** | **\|t\|** | **P value** | **P value summary** |
| β0 | Intercept | 1137 | 4160 | -7113 to 9386 | 0.2733 | 0.7852 | ns |
| β1 | Waist to Height Ratio | 3856 | 7321 | -10664 to 18377 | 0.5267 | 0.5995 | ns |

| **Supplementary Table S.4.4 Crude regression models for IL-6**  **(Waist to Height Ratio)** | | | | | | | |
| --- | --- | --- | --- | --- | --- | --- | --- |
| **Parameter estimates** | **Variable** | **Estimate** | **Standard error** | **95% CI (asymptotic)** | **\|t\|** | **P value** | **P value summary** |
| β0 | Intercept | 9591 | 3902 | 1852 to 17330 | 2.458 | 0.0156 | * |
| β1 | Waist to Height Ratio | -15556 | 6868 | -29177 to -1934 | 2.265 | 0.0256 | * |

| **Supplementary Table S.5.1 Adjusted regression models for adiponectin**  **(Body Mass Index)** | | | | | | | | | | | | | |
| --- | --- | --- | --- | --- | --- | --- | --- | --- | --- | --- | --- | --- | --- |
| **Parameter estimates** | **Variable** | | **Estimate** | | **Standard error** | | **95% CI (asymptotic)** | | **\|t\|** | | **P value** | | **P value summary** |
| β0 | Intercept | | 1001 | | 160,9 | | 681.7 to 1320 | | 6,224 | | <0.0001 | | **** |
| β1 | BMI | | 40,97 | | 55,86 | | -69.95 to 151.9 | | 0,7334 | | 0,4651 | | ns |
| β2 | Age | | 2,915 | | 1,647 | | -0.3550 to 6.185 | | 1,77 | | 0,08 | | ns |
| β3 | Gender | | -56,82 | | 57,36 | | -170.7 to 57.07 | | 0,9906 | | 0,3244 | | ns |
| β4 | Risk_AMI | | 33,52 | | 57,07 | | -79.80 to 146.8 | | 0,5873 | | 0,5584 | | ns |
| β5 | AMI type | | 121,6 | | 78,2 | | -33.70 to 276.8 | | 1,555 | | 0,1234 | | ns |
| β6 | LDL-c | | -0,4311 | | 0,5563 | | -1.536 to 0.6733 | | 0,7751 | | 0,4402 | | ns |
| β7 | Triglyceride | | 0,1121 | | 0,2678 | | -0.4197 to 0.6438 | | 0,4185 | | 0,6765 | | ns |
| β8 | HDL-c | | -4,311 | | 2,516 | | -9.307 to 0.6849 | | 1,713 | | 0,09 | | ns |
| β9 | Insulin | | 0,02682 | | 0,03175 | | -0.03621 to 0.08985 | | 0,845 | | 0,4003 | | ns |
| β10 | Creatinine | | 0,6677 | | 5,477 | | -10.21 to 11.54 | | 0,1219 | | 0,9032 | | ns |
| **Supplementary Table S.5.2 Adjusted regression models for FGF21**  **(Body Mass Index)** | | | | | | | | | | | | | |
| **Parameter estimates** | | **Variable** | | **Estimate** | | **Standard error** | | **95% CI (asymptotic)** | | **\|t\|** | **P value** | **P value summary** | |
| β0 | | Intercept | | 734,3 | | 621,1 | | -498.9 to 1968 | | 1,182 | 0,2401 | ns | |
| β1 | | BMI | | 264,4 | | 215,7 | | -163.9 to 692.6 | | 1,226 | 0,2234 | ns | |
| β2 | | Age | | 7,702 | | 6,359 | | -4.924 to 20.33 | | 1,211 | 0,2289 | ns | |
| β3 | | Gender | | 155,9 | | 221,5 | | -283.8 to 595.7 | | 0,7039 | 0,4832 | ns | |
| β4 | | Risk_AMI | | 409 | | 220,4 | | -28.53 to 846.6 | | 1,856 | 0,0666 | ns | |
| β5 | | AMI type | | -355,7 | | 302 | | -955.3 to 243.9 | | 1,178 | 0,2418 | ns | |
| β6 | | LDL-c | | 1,043 | | 2,148 | | -3.222 to 5.307 | | 0,4855 | 0,6285 | ns | |
| β7 | | Triglyceride | | -0,2567 | | 1,034 | | -2.310 to 1.796 | | 0,2483 | 0,8045 | ns | |
| β8 | | HDL-c | | -18,28 | | 9,715 | | -37.57 to 1.006 | | 1,882 | 0,0629 | ns | |
| β9 | | Insulin | | 0,02803 | | 0,1226 | | -0.2154 to 0.2714 | | 0,2286 | 0,8196 | ns | |
| β10 | | Creatinine | | 14,77 | | 21,15 | | -27.22 to 56.77 | | 0,6986 | 0,4865 | ns | |

| **Supplementary Table S.5.3 Adjusted regression models for GDF15**  **(Body Mass Index)** | | | | | | | |
| --- | --- | --- | --- | --- | --- | --- | --- |
| **Parameter estimates** | **Variable** | **Estimate** | **Standard error** | **95% CI (asymptotic)** | **\|t\|** | **P value** | **P value summary** |
| β0 | Intercept | 2329 | 3364 | -4351 to 9009 | 0,6923 | 0,4904 | ns |
| β1 | BMI | 55,42 | 1168 | -2264 to 2375 | 0,04744 | 0,9623 | ns |
| β2 | Age | 48,12 | 34,45 | -20.28 to 116.5 | 1,397 | 0,1657 | ns |
| β3 | Gender | 2997 | 1200 | 615.4 to 5380 | 2,498 | 0,0142 | * |
| β4 | Risk AMI | 157,5 | 1194 | -2213 to 2528 | 0,132 | 0,8953 | ns |
| β5 | AMI type | -379,3 | 1636 | -3627 to 2868 | 0,2319 | 0,8171 | ns |
| β6 | LDL-c | 4,077 | 11,63 | -19.02 to 27.18 | 0,3504 | 0,7268 | ns |
| β7 | Triglyceride | -2,476 | 5,602 | -13.60 to 8.646 | 0,4421 | 0,6595 | ns |
| β8 | HDL-c | -64,37 | 52,63 | -168.9 to 40.12 | 1,223 | 0,2243 | ns |
| β9 | Insulin | -0,5408 | 0,664 | -1.859 to 0.7775 | 0,8145 | 0,4174 | ns |
| β10 | Creatinine | -28,33 | 114,6 | -255.8 to 199.1 | 0,2473 | 0,8052 | ns |

| **Supplementary Table S.5.4 Adjusted regression models for IL-6**  **(Body Mass Index)** | | | | | | | |
| --- | --- | --- | --- | --- | --- | --- | --- |
| **Parameter estimates** | **Variable** | **Estimate** | **Standard error** | **95% CI (asymptotic)** | **\|t\|** | **P value** | **P value summary** |
| β0 | Intercept | 3808 | 3372 | -2887 to 10503 | 1,129 | 0,2616 | ns |
| β1 | BMI | -869,8 | 1171 | -3195 to 1455 | 0,7428 | 0,4595 | ns |
| β2 | Age | -1,714 | 34,52 | -70.26 to 66.83 | 0,04965 | 0,9605 | ns |
| β3 | Gender | 1194 | 1202 | -1193 to 3582 | 0,9933 | 0,3231 | ns |
| β4 | Risk AMI | -795,6 | 1196 | -3171 to 1580 | 0,665 | 0,5077 | ns |
| β5 | AMI type | 2254 | 1639 | -1001 to 5509 | 1,375 | 0,1723 | ns |
| β6 | LDL-c | -14,65 | 11,66 | -37.80 to 8.502 | 1,256 | 0,2121 | ns |
| β7 | Triglyceride | -1,09 | 5,614 | -12.24 to 10.06 | 0,1941 | 0,8465 | ns |
| β8 | HDL-c | -32,77 | 52,74 | -137.5 to 71.96 | 0,6212 | 0,536 | ns |
| β9 | Insulin | -0,3259 | 0,6655 | -1.647 to 0.9954 | 0,4898 | 0,6254 | ns |
| β10 | Creatinine | 12,62 | 114,8 | -215.4 to 240.6 | 0,1099 | 0,9127 | ns |

| **Supplementary Table S.6.1 Adjusted regression models for adiponectin**  **(Waist Circumference)** | | | | | | | |
| --- | --- | --- | --- | --- | --- | --- | --- |
| **Parameter estimates** | **Variable** | **Estimate** | **Standard error** | **95% CI (asymptotic)** | **\|t\|** | **P value** | **P value summary** |
| β0 | Intercept | 1480 | 274.8 | 934.4 to 2025 | 5.386 | <0.0001 | **** |
| β1 | Age | 2.913 | 1.61 | -0.2842 to 6.109 | 1.809 | 0.0736 | ns |
| β2 | Gender | -42.35 | 56.11 | -153.8 to 69.06 | 0.7547 | 0.4523 | ns |
| β3 | Risk_AMI | -31.18 | 53.08 | -136.6 to 74.20 | 0.5875 | 0.5583 | ns |
| β4 | AMI type | 142.3 | 58.73 | 25.65 to 258.9 | 2.422 | 0.0173 | * |
| β5 | LDL-c | -0.4263 | 0.5396 | -1.498 to 0.6452 | 0.79 | 0.4315 | ns |
| β6 | Triglyceride | 0.1393 | 0.2617 | -0.3802 to 0.6589 | 0.5325 | 0.5956 | ns |
| β7 | HDL-c | -3.773 | 2.471 | -8.680 to 1.134 | 1.527 | 0.1302 | ns |
| β8 | Insulin | 0.02084 | 0.03134 | -0.04139 to 0.08306 | 0.6648 | 0.5078 | ns |
| β9 | Creatinine | 1.634 | 5.378 | -9.043 to 12.31 | 0.3039 | 0.7619 | ns |
| β10 | Waist Circumference | -4.831 | 2.437 | -9.670 to 0.007017 | 1.983 | 0.0503 | ns |

| **Supplementary Table S.6.2 Adjusted regression models for FGF21**  **(Waist Circumference)** | | | | | | | |
| --- | --- | --- | --- | --- | --- | --- | --- |
| **Parameter estimates** | **Variable** | **Estimate** | **Standard error** | **95% CI (asymptotic)** | **\|t\|** | **P value** | **P value summary** |
| β0 | Intercept | 1684 | 1084 | -468.4 to 3837 | 1,554 | 0,1237 | ns |
| β1 | Age | 7,084 | 6,353 | -5.530 to 19.70 | 1,115 | 0,2677 | ns |
| β2 | Gender | 205,5 | 221,4 | -234.1 to 645.1 | 0,9283 | 0,3556 | ns |
| β3 | Risk_AMI | 197,6 | 209,4 | -218.2 to 613.4 | 0,9435 | 0,3479 | ns |
| β4 | AMI type | -142,4 | 231,7 | -602.5 to 317.7 | 0,6145 | 0,5403 | ns |
| β5 | LDL-c | 0,7161 | 2,129 | -3.512 to 4.944 | 0,3363 | 0,7374 | ns |
| β6 | Triglyceride | -0,1113 | 1,032 | -2.161 to 1.939 | 0,1078 | 0,9144 | ns |
| β7 | HDL-c | -16,64 | 9,751 | -36.00 to 2.722 | 1,706 | 0,0912 | ns |
| β8 | Insulin | 0,01811 | 0,1237 | -0.2274 to 0.2636 | 0,1465 | 0,8839 | ns |
| β9 | Creatinine | 17,82 | 21,22 | -24.31 to 59.95 | 0,8398 | 0,4032 | ns |
| β10 | Waist Circumference | -8,278 | 9,615 | -27.37 to 10.81 | 0,8609 | 0,3915 | ns |

| **Supplementary Table S.6.2 Adjusted regression models for GDF15**  **(Waist Circumference)** | | | | | | | |
| --- | --- | --- | --- | --- | --- | --- | --- |
| Parameter estimates | Variable | Estimate | Standard error | 95% CI (asymptotic) | \|t\| | P value | P value summary |
| β0 | Intercept | 10556 | 5760 | -880.7 to 21992 | 1,833 | 0,07 | ns |
| β1 | Age | 50,25 | 33,75 | -16.76 to 117.3 | 1,489 | 0,1399 | ns |
| β2 | Gender | 3172 | 1176 | 836.6 to 5508 | 2,697 | 0,0083 | ** |
| β3 | Risk AMI | -659,8 | 1113 | -2869 to 1549 | 0,5931 | 0,5546 | ns |
| β4 | AMI type | -633,8 | 1231 | -3078 to 1811 | 0,5148 | 0,6079 | ns |
| β5 | LDL-c | 5,351 | 11,31 | -17.11 to 27.81 | 0,473 | 0,6373 | ns |
| β6 | Triglyceride | -2,331 | 5,485 | -13.22 to 8.560 | 0,425 | 0,6718 | ns |
| β7 | HDL-c | -57,17 | 51,81 | -160.0 to 45.69 | 1,104 | 0,2726 | ns |
| β8 | Insulin | -0,6507 | 0,657 | -1.955 to 0.6538 | 0,9904 | 0,3245 | ns |
| β9 | Creatinine | -15,71 | 112,7 | -239.5 to 208.1 | 0,1394 | 0,8894 | ns |
| β10 | Waist Circumference | -87,63 | 51,08 | -189.1 to 13.79 | 1,716 | 0,0895 | ns |

| **Supplementary Table S.6.2 Adjusted regression models for IL-6**  **(Waist Circumference)** | | | | | | | |
| --- | --- | --- | --- | --- | --- | --- | --- |
| **Parameter estimates** | **Variable** | **Estimate** | **Standard error** | **95% CI (asymptotic)** | **\|t\|** | **P value** | **P value summary** |
| β0 | Intercept | 10776 | 5804 | -748.6 to 22300 | 1,857 | 0,0665 | ns |
| β1 | Age | 3,167 | 34,01 | -64.36 to 70.69 | 0,09313 | 0,926 | ns |
| β2 | Gender | 1238 | 1185 | -1116 to 3591 | 1,044 | 0,2991 | ns |
| β3 | Risk AMI | -1072 | 1121 | -3298 to 1154 | 0,956 | 0,3415 | ns |
| β4 | AMI type | 1177 | 1241 | -1287 to 3640 | 0,9484 | 0,3454 | ns |
| β5 | LDL-c | -11,89 | 11,4 | -34.52 to 10.74 | 1,043 | 0,2996 | ns |
| β6 | Triglyceride | -1,424 | 5,527 | -12.40 to 9.550 | 0,2577 | 0,7972 | ns |
| β7 | HDL-c | -29,56 | 52,2 | -133.2 to 74.09 | 0,5662 | 0,5726 | ns |
| β8 | Insulin | -0,4288 | 0,662 | -1.743 to 0.8857 | 0,6477 | 0,5188 | ns |
| β9 | Creatinine | 17,66 | 113,6 | -207.9 to 243.2 | 0,1554 | 0,8768 | ns |
| β10 | Waist Circumference | -80,76 | 51,47 | -183.0 to 21.44 | 1,569 | 0,12 | ns |

| **Supplementary Table S.7.1 Adjusted regression models for adiponectin**  **(Body Fat)** | | | | | | | |
| --- | --- | --- | --- | --- | --- | --- | --- |
| **Parameter estimates** | **Variable** | **Estimate** | **Standard error** | **95% CI (asymptotic)** | **\|t\|** | **P value** | **P value summary** |
| β0 | Intercept | 1062 | 158,4 | 747.7 to 1377 | 6,705 | <0.0001 | **** |
| β1 | Age | 2,672 | 1,633 | -0.5698 to 5.915 | 1,637 | 0,1051 | ns |
| β2 | Gender | -62,77 | 57,45 | -176.8 to 51.31 | 1,093 | 0,2774 | ns |
| β3 | Risk_AMI | 0,4595 | 50 | -98.81 to 99.73 | 0,009191 | 0,9927 | ns |
| β4 | AMI type | 153,4 | 59,08 | 36.12 to 270.7 | 2,597 | 0,0109 | * |
| β5 | LDL-c | -0,3792 | 0,5553 | -1.482 to 0.7233 | 0,6829 | 0,4963 | ns |
| β6 | Triglyceride | 0,149 | 0,2655 | -0.3781 to 0.6761 | 0,5613 | 0,5759 | ns |
| β7 | HDL-c | -3,033 | 2,673 | -8.341 to 2.275 | 1,135 | 0,2594 | ns |
| β8 | Insulin | 0,02311 | 0,03177 | -0.03997 to 0.08618 | 0,7274 | 0,4688 | ns |
| β9 | Creatinine | 1,686 | 5,473 | -9.181 to 12.55 | 0,3081 | 0,7587 | ns |
| β10 | Body Fat | -2,919 | 2,482 | -7.848 to 2.009 | 1,176 | 0,2425 | ns |

| **Supplementary Table S.7.2 Adjusted regression models for FGF21**  **(Body Fat)** | | | | | | | |
| --- | --- | --- | --- | --- | --- | --- | --- |
| **Parameter estimates** | **Variable** | **Estimate** | **Standard error** | **95% CI (asymptotic)** | **\|t\|** | **P value** | **P value summary** |
| β0 | Intercept | 814,8 | 616,9 | -410.1 to 2040 | 1,321 | 0,1898 | ns |
| β1 | Age | 7,185 | 6,359 | -5.441 to 19.81 | 1,13 | 0,2614 | ns |
| β2 | Gender | 221,3 | 223,7 | -222.9 to 665.6 | 0,9893 | 0,325 | ns |
| β3 | Risk AMI | 305,6 | 194,7 | -81.00 to 692.2 | 1,569 | 0,1199 | ns |
| β4 | AMI type | -97,54 | 230,1 | -554.4 to 359.3 | 0,4239 | 0,6726 | ns |
| β5 | LDL-c | 0,2397 | 2,162 | -4.054 to 4.533 | 0,1109 | 0,912 | ns |
| β6 | Triglyceride | -0,1679 | 1,034 | -2.221 to 1.885 | 0,1624 | 0,8713 | ns |
| β7 | HDL-c | -20,49 | 10,41 | -41.16 to 0.1859 | 1,968 | 0,0521 | ns |
| β8 | Insulin | 0,03922 | 0,1237 | -0.2064 to 0.2849 | 0,3171 | 0,7519 | ns |
| β9 | Creatinine | 14,61 | 21,31 | -27.71 to 56.93 | 0,6856 | 0,4947 | ns |
| β10 | Body Fat | 8,265 | 9,667 | -10.93 to 27.46 | 0,8549 | 0,3948 | ns |

| **Supplementary Table S.7.3 Adjusted regression models for GDF15**  **(Body Fat)** | | | | | | | |
| --- | --- | --- | --- | --- | --- | --- | --- |
| **Parameter estimates** | **Variable** | **Estimate** | **Standard error** | **95% CI (asymptotic)** | **\|t\|** | **P value** | **P value summary** |
| β0 | Intercept | 2536 | 3327 | -4070 to 9142 | 0,7623 | 0,4478 | ns |
| β1 | Age | 47,38 | 34,29 | -20.71 to 115.5 | 1,382 | 0,1704 | ns |
| β2 | Gender | 2948 | 1207 | 552.8 to 5344 | 2,444 | 0,0164 | * |
| β3 | Risk AMI | 69,4 | 1050 | -2015 to 2154 | 0,0661 | 0,9474 | ns |
| β4 | AMI type | -357 | 1241 | -2821 to 2107 | 0,2877 | 0,7742 | ns |
| β5 | LDL-c | 4,597 | 11,66 | -18.56 to 27.75 | 0,3942 | 0,6943 | ns |
| β6 | Triglyceride | -2,367 | 5,575 | -13.44 to 8.702 | 0,4246 | 0,6721 | ns |
| β7 | HDL-c | -58,52 | 56,14 | -170.0 to 52.96 | 1,042 | 0,3 | ns |
| β8 | Insulin | -0,5598 | 0,6672 | -1.884 to 0.7649 | 0,839 | 0,4036 | ns |
| β9 | Creatinine | -24,29 | 114,9 | -252.5 to 203.9 | 0,2114 | 0,8331 | ns |
| β10 | Body Fat | -14,65 | 52,13 | -118.2 to 88.86 | 0,281 | 0,7793 | ns |

| **Supplementary Table S.7.4 Adjusted regression models for IL-6**  **(Body Fat)** | | | | | | | |
| --- | --- | --- | --- | --- | --- | --- | --- |
| **Parameter estimates** | **Variable** | **Estimate** | **Standard error** | **95% CI (asymptotic)** | **\|t\|** | **P value** | **P value summary** |
| β0 | Intercept | 3468 | 3343 | -3169 to 10105 | 1,037 | 0,3022 | ns |
| β1 | Age | 0,2393 | 34,46 | -68.17 to 68.65 | 0,006945 | 0,9945 | ns |
| β2 | Gender | 1004 | 1212 | -1403 to 3411 | 0,8282 | 0,4097 | ns |
| β3 | Risk AMI | -428,9 | 1055 | -2524 to 1666 | 0,4066 | 0,6853 | ns |
| β4 | AMI type | 1418 | 1247 | -1057 to 3893 | 1,137 | 0,2583 | ns |
| β5 | LDL-c | -12,28 | 11,72 | -35.55 to 10.98 | 1,048 | 0,2972 | ns |
| β6 | Triglyceride | -1,418 | 5,602 | -12.54 to 9.704 | 0,2531 | 0,8007 | ns |
| β7 | HDL-c | -28,03 | 56,41 | -140.0 to 83.97 | 0,497 | 0,6204 | ns |
| β8 | Insulin | -0,3543 | 0,6703 | -1.685 to 0.9766 | 0,5286 | 0,5984 | ns |
| β9 | Creatinine | 11,53 | 115,5 | -217.8 to 240.8 | 0,09987 | 0,9207 | ns |
| β10 | Body Fat | -20,68 | 52,38 | -124.7 to 83.32 | 0,3949 | 0,6938 | ns |

| **Supplementary Table S.8.1 Adjusted regression models for adiponectin**  **(Waist to Height Ratio)** | | | | | | | |
| --- | --- | --- | --- | --- | --- | --- | --- |
| **Parameter estimates** | **Variable** | **Estimate** | **Standard error** | **95% CI (asymptotic)** | **\|t\|** | **P value** | **P value summary** |
| β0 | Intercept | 1377 | 255,7 | 869.2 to 1885 | 5,384 | <0.0001 | **** |
| β1 | Age | 3,3 | 1,645 | 0.03436 to 6.566 | 2,006 | 0,0477 | * |
| β2 | Gender | -51,29 | 56,21 | -162.9 to 60.31 | 0,9126 | 0,3638 | ns |
| β3 | Risk_AMI | -15,02 | 51,14 | -116.6 to 86.52 | 0,2938 | 0,7696 | ns |
| β4 | AMI type | 149,6 | 58,68 | 33.12 to 266.1 | 2,55 | 0,0124 | * |
| β5 | LDL-c | -0,4201 | 0,5432 | -1.499 to 0.6584 | 0,7733 | 0,4413 | ns |
| β6 | Triglyceride | 0,1376 | 0,263 | -0.3846 to 0.6598 | 0,523 | 0,6022 | ns |
| β7 | HDL-c | -4,184 | 2,476 | -9.101 to 0.7327 | 1,69 | 0,0944 | ns |
| β8 | Insulin | 0,02963 | 0,03139 | -0.03270 to 0.09196 | 0,9438 | 0,3477 | ns |
| β9 | Creatinine | 0,6351 | 5,398 | -10.08 to 11.35 | 0,1177 | 0,9066 | ns |
| β10 | Waist to Height Ratio | -648,1 | 378,9 | -1401 to 104.3 | 1,71 | 0,0905 | ns |

| **Supplementary Table S.8.2 Adjusted regression models for FGF21**  **(Waist to Height Ratio)** | | | | | | | |
| --- | --- | --- | --- | --- | --- | --- | --- |
| **Parameter estimates** | **Variable** | **Estimate** | **Standard error** | **95% CI (asymptotic)** | **\|t\|** | **P value** | **P value summary** |
| β0 | Intercept | -3065 | 5370 | -13728 to 7597 | 0,5708 | 0,5695 | ns |
| β1 | Age | 39,92 | 34,54 | -28.67 to 108.5 | 1,156 | 0,2508 | ns |
| β2 | Gender | 3000 | 1180 | 656.4 to 5344 | 2,542 | 0,0127 | * |
| β3 | Risk AMI | 554,6 | 1074 | -1578 to 2687 | 0,5164 | 0,6068 | ns |
| β4 | AMI type | -180,9 | 1232 | -2627 to 2266 | 0,1468 | 0,8836 | ns |
| β5 | LDL-c | 2,708 | 11,41 | -19.94 to 25.36 | 0,2374 | 0,8129 | ns |
| β6 | Triglyceride | -2,521 | 5,523 | -13.49 to 8.446 | 0,4564 | 0,6492 | ns |
| β7 | HDL-c | -63,76 | 52 | -167.0 to 39.50 | 1,226 | 0,2233 | ns |
| β8 | Insulin | -0,5833 | 0,6592 | -1.892 to 0.7256 | 0,8849 | 0,3785 | ns |
| β9 | Creatinine | -22,86 | 113,4 | -247.9 to 202.2 | 0,2016 | 0,8406 | ns |
| β10 | Waist to Height Ratio | 10102 | 7958 | -5698 to 25902 | 1,269 | 0,2074 | ns |

| **Supplementary Table S.8.3 Adjusted regression models for GDF15**  **(Waist to Height Ratio)** | | | | | | | |
| --- | --- | --- | --- | --- | --- | --- | --- |
| **Parameter estimates** | **Variable** | **Estimate** | **Standard error** | **95% CI (asymptotic)** | **\|t\|** | **P value** | **P value summary** |
| β0 | Intercept | 249,9 | 1004 | -1744 to 2244 | 0,2489 | 0,804 | ns |
| β1 | Age | 5,889 | 6,459 | -6.935 to 18.71 | 0,9118 | 0,3642 | ns |
| β2 | Gender | 189,1 | 220,7 | -249.1 to 627.4 | 0,857 | 0,3936 | ns |
| β3 | Risk AMI | 323,9 | 200,8 | -74.84 to 722.6 | 1,613 | 0,1101 | ns |
| β4 | AMI type | -95,61 | 230,4 | -553.1 to 361.9 | 0,415 | 0,6791 | ns |
| β5 | LDL-c | 0,4318 | 2,133 | -3.803 to 4.667 | 0,2024 | 0,84 | ns |
| β6 | Triglycerides | -0,1312 | 1,033 | -2.182 to 1.919 | 0,127 | 0,8992 | ns |
| β7 | HDL-c | -17,25 | 9,724 | -36.56 to 2.057 | 1,774 | 0,0793 | ns |
| β8 | Insulin | 0,02331 | 0,1233 | -0.2214 to 0.2681 | 0,1891 | 0,8504 | ns |
| β9 | Creatinine | 17,28 | 21,2 | -24.80 to 59.37 | 0,8154 | 0,4169 | ns |
| β10 | Waist to Height Ratio | 1229 | 1488 | -1726 to 4183 | 0,8259 | 0,411 | ns |

| **Supplementary Table S.8.4 Adjusted regression models for IL-6**  **(Waist to Height Ratio)** | | | | | | | |
| --- | --- | --- | --- | --- | --- | --- | --- |
| **Parameter estimates** | **Variable** | **Estimate** | **Standard error** | **95% CI (asymptotic)** | **\|t\|** | **P value** | **P value summary** |
| β0 | Intercept | 10327 | 5366 | -326.9 to 20981 | 1,925 | 0,0573 | ns |
| β1 | Age | 11,53 | 34,51 | -57.00 to 80.06 | 0,3341 | 0,7391 | ns |
| β2 | Gender | 1089 | 1179 | -1253 to 3431 | 0,9235 | 0,3581 | ns |
| β3 | Risk AMI | -901,5 | 1073 | -3032 to 1229 | 0,8402 | 0,403 | ns |
| β4 | AMI type | 1265 | 1231 | -1180 to 3709 | 1,027 | 0,3069 | ns |
| β5 | LDL-c | -11,49 | 11,4 | -34.12 to 11.14 | 1,008 | 0,3162 | ns |
| β6 | triglycerides | -1,437 | 5,519 | -12.39 to 9.520 | 0,2604 | 0,7951 | ns |
| β7 | HDL-c | -36,53 | 51,96 | -139.7 to 66.64 | 0,7031 | 0,4837 | ns |
| β8 | Insulin | -0,2718 | 0,6587 | -1.580 to 1.036 | 0,4126 | 0,6808 | ns |
| β9 | Creatinine | -0,2391 | 113,3 | -225.1 to 224.7 | 0,002111 | 0,9983 | ns |
| β10 | Waist to Height Ratio | -13203 | 7951 | -28990 to 2584 | 1,66 | 0,1001 | ns |

**Supplementary Table S9. Circulating adipokine and inflammatory cytokine levels in non-obese and obese patients with acute myocardial infarction**

| Variables | Non-obese (n=62) | Obese (n=43) | Healthy Controls (n=10) | *p value* |
| --- | --- | --- | --- | --- |
| Adiponectin (ng/mL), Mean±SD | 1147 ± 208.3 | 1001 ± 131 | 1047 ± 86.57 | <0,0001 |
| FGF-21 (pg/mL), Median (min-max) | 870 (205-2589) | 536 (195-2656) | 231 (132-284) | 0,0151 |
| GDF-15 (pg/mL), Median (min-max) | 2285 (882-26075) | 1747 (915-9959) | 947 (660-2173) | 0.0232 |
| IL-6 (pg/mL), Median (min-max) | 19.85 (7.95-34734) | 14.56 (6.75-2718) | 14.56 (9.13-211.5) | 0.0373 |

Data are presented as mean ± SD or median (min–max). p values were calculated using the independent t-test or Mann–Whitney U test, as appropriate, for non-obese versus obese AMI comparisons. Healthy controls are shown only as descriptive reference values and were not included in hypothesis testing. Statistical significance was set at p < 0.05.
